# Treatment Personalization and Precision Mental Health Care: Where are we and where do we want to go?

**DOI:** 10.1007/s10488-024-01407-w

**Published:** 2024-08-22

**Authors:** Danilo Moggia, Wolfgang Lutz, Eva-Lotta Brakemeier, Leonard Bickman

**Affiliations:** 1 University of Trier, Trier, Germany; 2 University of Greifswald, Greifswald, Germany; 3 Vanderbilt University, Nashville, USA

The concept of personalizing mental health interventions to align with individual patients’ unique characteristics, needs, and circumstances has been a topic of longstanding interest in the field (e.g., Howard et al., 1996; Lambert et al., 2001; Paul, 1967). In fact, personalizing treatments is likely inherent to the practice of every clinician who engages in a clinical decision-making process when confronted with a patient to determine an appropriate treatment plan that considers the individual’s features. However, the specific methods and information sources employed by clinicians in this process vary considerably. Clinical intuition probably represents the most commonly utilized resources for making these decisions (Cohen et al., 2021).

## Challenges of Clinical Intuition

It is well recognized that clinical intuition tends to be fallible and frequently falls short of the performance achieved by data-driven classification and prognostic models (Ægisdóttir et al., 2006; Lilienfeld & Lynn, 2014). To enhance clinical decision-making, various research concepts have emerged, including routine outcome monitoring (ROM), measurement-based care, and patient-focused research. Patient-focused research encompasses routine outcome measurements and the provision of psychometric feedback to therapists and patients throughout treatment. It leverages clinical population data to develop insights and tools that can inform treatment decisions to personalize treatments at an individual level. These tools aim to monitor treatment progress and provide feedback to therapists and patients to make adjustments to enhance treatment outcomes (see The Use of Feedback in Mental Health Services. *Administration and Policy in Mental Health and Mental Health Services*. Volume 52, Issue 1, 2025).

## Personalization and Advancements in Data-Informed Approaches

Nevertheless, with the advent of big data, machine learning, and artificial intelligence in recent years, personalization has moved towards the concept of precision mental health care (Bickman, 2020; Bickman et al., 2016; Lutz et al., 2021, 2022).

Precision mental health care emphasizes using personalized data-driven strategies to optimize outcomes for a particular patient. This approach integrates various forms of collecting data on patients’ symptoms and behaviors (i.e., measurement-based care approach) using digital tools, such as digital questionnaires, mobile phone apps, and wearable devices. This data is compared with large datasets of already treated patients through algorithms that can provide a personalized clinical recommendation, which can be used to inform treatment decisions (Chekroud et al., 2021; Lutz et al., 2019). In this way, precision mental health care deals with the vast heterogeneity of patients’ features and the significant variability in the effectiveness of different mental health treatments for different individuals (Feczko et al., 2019; Kessler et al., 2017). By better identifying the specific prognostic factors and moderators that are associated with particular treatment outcomes for specific patients’ profiles or subpopulations, precision mental health care can help clinicians identify the most effective treatment for the individual patient and improve their outcomes (Bickman, 2020).

## Role of Algorithms and Clinical Decision Support

To achieve the goals of precision mental healthcare, algorithms should be integrated into clinical decision support systems that are user-friendly and accessible to clinicians (Sutton et al., 2020). The aim of incorporating algorithms is not to replace the crucial role of clinicians and the human relationship involved in the healthcare process, but to provide them with digital support and evidence-based clinical recommendations that are valid, reliable, and precise. In this sense, it is important to ensure that these algorithms are implemented to support clinical decision-making and the therapeutic relationship without hindering them. Ultimately, successfully implementing algorithms in clinical decision support systems can help clinicians provide better care.

## Integration into the Mental Health Care Process

Precision mental health care initiatives can be seamlessly integrated into the entire healthcare process, spanning from prevention to diagnosis, patient-clinician matching, treatment selection, monitoring, and adaptation (Delgadillo & Lutz, 2020). The articles in this Special Issue cover various initiatives related to different stages of the healthcare process at different stages of development. For instance, we count on studies that go from identifying the patients’ needs that should be considered and assessed when developing precision mental health care initiatives (precursors of personalization), passing by those that present quantitative methods for developing them, developed predictive models, deal with therapist effects and patient-clinician matching and evaluate the implementation of such tools, to a paper that present a general framework for precision mental health care and a comprehensive clinical decision support system. All the papers represent the culmination of an extensive and rigorous selection process, with contributions from some of the most prominent scholars and experts in the field.

## Articles in This Special Issue

Gryesten et al. (2023) present a qualitative study that explored patients’ and therapists’ needs in group cognitive-behavioral therapy (GCBT). They argue about the challenge of personalizing psychotherapy for individual patients in group settings, especially for patients who are not responding as expected (according to routine outcome monitoring). With phenomenological-hermeneutic analysis, they identified five themes representing different patients’ needs: (1) Individual attention from the therapists; (2) Exploring individual patients’ experiences and psychological mechanisms; (3) Focus on the patient’s life outside therapy; (4) Individualized extended assessment; (5) Individualized agreement on the therapeutic tasks. They show that patients have different needs when they are not making progress in therapy, and these needs, when unmet, can negatively impact the experience of GCBT. Finally, the authors give recommendations on how to personalize psychotherapy in the context of GCBT.

Sheikh et al. (2024) present also a qualitative study addressing the question of what personalized mental health support should involve according to the perspective of young people with lived experience and professionals from eight countries. Using the rigorous and accelerated data reduction (RADaR) technique, the authors found 11 themes the participants referred to as topics that should be considered to personalize treatments for young people. These themes were classified following Brofrenbrenner’s ecological theory of development, grouping them at the micro level (e.g., understanding and accepting yourself), meso level (e.g., understanding and acceptance from others), and macro level (e.g., promoting equity and decreasing discrimination and social injustice). Participants agreed that for support to be effective, it must be tailored to the needs of the individual. It is, therefore, crucial to tailor support to each young person, enabling them to choose the most appropriate options and have a say in their care. In addition, it is important to consider the links between individual preferences and circumstances when considering personalizing mental health support.

Scholten et al. (2024) outline a step-by-step implementation protocol for a single-case outpatient clinic. The authors explain how they will implement a single-case experimental design infrastructure combined with experience sampling methods (ESM) to extend routine outcome monitoring further. This infrastructure will allow longitudinal data collection and systematic manipulation to advance personalization efforts further. They plan to develop, implement, and evaluate the infrastructure for single-case experimental design in six successive studies involving stakeholders. During the project identification phase (Study 1), a business model for the clinic infrastructure will be developed that aligns its vision with its broader context. The infrastructure prototype is defined in the development phase (Studies 2 and 3), including elements such as the single-case experimental design procedure, the ESM protocol, and the ESM surveys. In the optimization phase (Study 4), the infrastructure will be rigorously tested to assess its feasibility and acceptability, and necessary adjustments will be made accordingly. In the evaluation phase, a pilot implementation study is conducted (Study 5), followed by full implementation using an intra-institutional A-B design (Study 6). Finally, the sustainability phase will ensure continuous monitoring and improvement of the infrastructure, with the added dimension of exploring the potential utility of the collected data in addressing questions of personalized psychotherapy research.

Blackwell (2024) introduces a novel design to test personalized psychological therapies embedded within routine practice, the “leapfrog” trial. The paper explains the value of adaptive platform trials (APT) in developing and testing personalized interventions. APT allows multiple interventions to be compared in a continuous manner, with interventions entering and exiting the trial based on a predefined decision algorithm. In this context, the leapfrog trial allows making multiple sequential comparisons against the most successful treatment so far using Bayesian statistics. This approach offers advantages in terms of reduced sample size requirements and increased efficiency in selecting the optimal treatment for specific patient subpopulations. The author presents a simulation study to show the approach and exemplify its advantages. Finally, he discusses how such trials might be implemented in routine practice, including potential challenges and limitations, as well as a long-term perspective on the development of personalized psychological therapies.

Salditt et al. (2023) present a tutorial on predicting heterogeneous treatment effects with meta-learners. Meta-learners are algorithms that decompose treatment effect estimation into multiple prediction tasks, each of which can be solved by any machine learning model. Meta-learners can evaluate which covariates drive treatment effect heterogeneity and predict individual treatment effects for new patients to derive personalized treatment recommendations. The authors show a data example of how most common meta-learners can be implemented in R, pointing out their usefulness and applications in psychotherapy research. Finally, they explain how heterogeneous treatment effects can be analyzed and pose some challenges in implementing meta-learners for precision mental health care research.

De Boer et al. (2024) outline a study aimed at predicting the disruptive behavior of patients in acute psychiatric units. They point out that it is critical to identify and understand early warning signs to prevent a disruptive crisis in a patient, with evidence suggesting that changes in a patient’s overall functioning may serve as indicators. Data from 931 patients during the first 28 days of hospitalization were analyzed using survival and Cox regression analyses. Disruptive behavior was predominantly observed in the first days of hospitalization. Patients with consistently stable, poor, or worsening overall functioning were at the highest risk for disruptive behavior. Improvement in overall functioning was associated with a decreased risk of disruptive behavior. It was possible to predict disruptive behavior two days before it manifested. The authors conclude that more attention is needed for increased focus on early interventions related to general functioning to prevent disruptive behavior.

Fernández-Álvarez et al. (2024) report a study to predict change trajectories from patients’ baseline characteristics. A sample of 257 patients attending an integrative psychotherapy network of psychotherapists in Buenos Aires, Argentina, was recruited. Using stepwise regression, six variables were selected to predict patients’ trajectories: social support, subjective distress, clinical distress, unemployment, sociocultural levels, and reactance level. Only social support, subjective distress, and clinical distress remained significant predictors when these variables were included in a hierarchical linear model. The authors discuss their findings and compare them with previous literature. They conclude that the results regarding social support are consistent with the literature, while unemployment and sociocultural levels move in the opposite direction compared to the available evidence.

Janse et al. (2024) report a study about early changes in patients receiving treatment for personality disorders. They analyzed changes in psychological distress and different personality domains (i.e., social attunement, relational functioning, identity integration, responsibility, self-control) finding that early changes in a specific domain were the strongest predictor of post-treatment outcome for that same domain. In addition, early changes in psychological distress significantly predicted outcomes in relational functioning, identity integration, and self-control. The authors conclude that monitoring early changes in specific personality domains beyond general psychological distress may help assess progress in treating patients with personality disorders.

Janse et al. (2024a, b) present an observational and naturalistic study of therapist effects. A sample of 68 therapists seeing 5,582 patients in a large mental health center was analyzed using hierarchical linear models to identify therapist effects. Several outcome measures were considered (i.e., psychological distress, attrition rates, referrals to other clinics, treatment duration, and patient satisfaction). Hierarchical cluster analysis was used to identify clusters of therapists defined by their performance on various outcome measures. Differences in therapist characteristics across clusters were analyzed. Therapist effects varied across outcomes, ranging from small to moderate. Four clusters of therapists with specific outcome profiles were identified (demonstrating effectiveness in some areas but not others). Some clusters differed in the proportion of master’s level psychologists and therapists in training. The authors conclude that therapists have different effectiveness profiles, supporting previous research showing that the most effective therapists are not necessarily the most experienced and spend more time in post-master’s training.

Building on previous research on patient-therapist matching, Coyne et al. (2024) introduce a study examining therapist characteristics that moderate the matching effect. In other words, for which therapists was the matching particularly important in achieving more effective outcomes? They examined three therapist moderators: effectiveness “spread” (i.e., greater variability in performance across patients’ presenting problems), overestimation of their measurement-based and problem-specific effectiveness, and the frequency with which they use routine patient-reported outcome monitoring in their practice. Using hierarchical linear models, they found that none of these characteristics moderated the match effect. However, the matching effect was larger for therapists with higher caseloads of more severe patients. The authors conclude that measurement-based matching may be particularly important for therapists who consistently treat patients with more severe symptoms and impairments.

Boswell et al. (2023) outline a qualitative study exploring the perspectives of patients and therapists on a patient-therapist matching algorithm in the context of measurement-based care. They combined methods from consensual qualitative research and grounded theory. They found that patients are comfortable selecting a provider (i.e., a therapist) based on a list of empirically well-matched recommendations. Patients showed interest in accessing more specific provider information online and that both provider outcome track records and therapist characteristics should be considered in the therapist selection process. Therapists pointed out that outcome data would be useful for matching patients to providers. Nevertheless, they also remarked that outcome data should not be the only factor used in provider selection. All therapists expressed a willingness to be included in preferred provider lists that incorporate track record data. The authors conclude that patients and therapists held.

positive views toward using therapist effectiveness data to help personalize mental health care. Additionally, patients and therapists acknowledged that other personalization factors should be considered alongside these data.

Finally, Lutz et al. (2023) state a general framework about precision mental health care, underscoring the progress in outcome measurement and data-driven decision support tools for therapists in psychological treatments over the past two decades. They point out that technological advancements, particularly computerized data assessment and feedback tools, have facilitated the widespread adoption of these approaches in various contexts. The paper highlights recent efforts to enhance the integration of clinical practice with precision tools, exemplified by clinicians’ use of psychometric data. This data helps therapists select therapeutic programs, strategies, and modules and enables real-time monitoring of a patient’s response to therapy. The authors present a data-informed decision support system, the Trier Treatment Navigator (TTN), designed to tailor individual psychological interventions to specific patient needs. Finally, they discuss the implications of implementing precision mental healthcare initiatives in clinical practice.

## Conclusion

The contributions in this Special Issue underscore the significant advancements in personalization and precision mental health care. These papers collectively highlight innovative methodologies, diverse patient populations, and a variety of mental health conditions, offering a comprehensive view of the current landscape. Nevertheless, despite these promising developments, several challenges remain unaddressed. Further research is needed to externally validate and refine predictive models, and ensure equitable access to personalized care through correct implementation (Deisenhofer et al., 2024). Predictive models should move in the direction of making recommendations based on causal mechanisms rather than mere associations between variables (Saxe et al., 2022), and more prospective studies testing the efficacy and effectiveness of these initiatives (in contrast to non-personalized treatments) should be performed (e.g., Delgadillo et al., 2022; Lutz et al., 2021; Nye et al., 2023). The findings presented here showcase the strides made and emphasize the necessity for continued exploration and development in this burgeoning field.

Overall, precision mental health care represents a promising new direction in mental health treatments that has the potential to improve patient outcomes and reduce the burden of mental health problems on individuals and society as a whole.

We hope this special issue will be a valuable resource for our readers and provide new insights and perspectives on a fascinating and innovative research field.
